# The inflammatory prognostic index as a potential predictor of prognosis in metastatic gastric cancer

**DOI:** 10.1038/s41598-023-34778-5

**Published:** 2023-05-12

**Authors:** Ahmet Ozveren, Atike Pinar Erdogan, Ferhat Ekinci

**Affiliations:** 1 Medical Oncology Department, Internal Medicine, Izmir Private Kent Hospital, Izmir, Turkey; 2 Medical Oncology Department, Internal Medicine, Faculty of Medicine, Manisa Celal Bayar University, Manisa, Turkey

**Keywords:** Cancer, Oncology

## Abstract

Clinical studies aimed at identifying effective and simple prognostic markers for gastric cancer are still being carried out. Inflammatory prognostic index (IPI) is being recognized as a promising prognostic marker in patients with Non-Small Cell Lung Cancer. To evaluate the prognostic utility of IPI in stage 4 gastric cancer. A total of 152 patients with stage 4 gastric cancer, whose laboratory parameters, progression-free survival (PFS) and overall survival (OS) data could be accessed, were evaluated. Kaplan Meier analysis was used for survival analyses. Hazard ratios were expressed with 95% CI values. All methods were performed in accordance with the relevant guidelines and regulations. Study was approved by the Manisa Celal Bayar University’s Non-Invasive Clinical Research Ethics Committee (approval No. E-85252386-050.04.04-49119, date: 22.03.2021). We confirm that all methods were performed in accordance with relevant named guidelines and regulations. Median age at diagnosis was 63 years (range: 32–88). The number of patients who received first-line chemotherapy was 129 (84.9%). Median PFS with first-line treatment was 5.3 months, while it was 3.3 months with second-line treatment. Median OS was 9.4 months. Median IPI score was 22.2. We evaluated IPI score for its value in detecting survival status with ROC analysis and identified an IPI cut-off score of 14.6. Low IPI score was significantly associated with longer PFS and OS compared to high IPI (PFS in high vs. low IPI, 3.6 vs. 7 months; *p* < 0.001) (OS in high vs. low IPI, 6.6 vs. 14.2 months; *p* < 0.001). IPI score can be an independent prognostic index that is inexpensive, easy to access and evaluate for patients with metastatic gastric cancer, and may be useful in predicting survival in daily practice.

## Introduction

Gastric cancer is the 5th most common cancer and the 3rd most common cause of cancer-related death^1,2^. Cancer-associated systemic inflammatory response is triggered by tumor microenvironment and is associated with tumor development, invasion, and metastasis^3^. Systemic inflammatory response is associated with predisposition to cachexia and deterioration of the patient’s performance^4^. Some inflammatory biomarkers generated using hematological and biochemical parameters have value in predicting adverse effects such as decreased overall or progression-free survival (OS, PFS) or resistance to chemotherapy, and thus, inflammatory biomarkers have been investigated in a number of tumor types^5,6^. The link between inflammation and cancer is very strong and just as complex. The interaction of cells responsible for inflammation and tumor cells varies according to suppressors or activators at various steps. Inflammation plays an important function in tumor development, invasion and metastasis. C-reactive protein (CRP), which is produced by liver cells, is an acute phase protein regulated by IL-1, IL 6 and tumor necrosis factor^7^. Many studies have shown that increased CRP is negatively associated with prognosis. Also, neutrophil/lymphocyte ratio (NLR) has been associated with poor prognosis in various cancers, and some studies have confirmed that NLR is a reliable marker in this respect.

Significant variation in these associations has been observed, and the sources of this variation are poorly understood. NLR may be demonstrative of the balance between the harmful effects of neutrophils and the beneficial effects of adaptive immunity^8^. Nonetheless, the prognostic potential of NLR may not be the same in all patient subgroups and in all solid tumors^9^.

Clinical studies aimed at determining effective and simple prognostic markers for gastric cancer are still being carried out. Various combinations of parameters, such as neutrophil-to-lymphocyte ratio (NLR), platelet-to-lymphocyte ratio (PLR) or the Glasgow prognostic score, have been used^10,11^. Subsequently, increasing evidence shows that prognostic markers can be used to discern prognosis in various malignant tumors. Inflammatory prognostic index (IPI), a measure based on CRP, NLR and serum albumin, has emerged as a promising prognostic marker in patients with Non-Small Cell Lung Cancer^12^.

In our study, we aimed to investigate whether IPI values are associated with PFS and OS in patients with stage-4 gastric cancer.

## Methodology

### Patients and methods

In our study, patients who were diagnosed with metastatic gastric cancer between 2013 and 2020 at Manisa Celal Bayar University Hospital and Private İzmir Kent Hospital were retrospectively analyzed. A total of 152 patients with stage 4 gastric cancer whose laboratory parameters, PFS data and OS data could be accessed, were evaluated. The blood tests of the patients were performed in the laboratory of the institute where they were treated. Pre-treatment parameters were addressed so that the administered treatments did not affect laboratory and performance results. The first blood tests were obtained before the treatment. Laboratory parameters, performance status, age at diagnosis, sex and treatments of the patients who applied to the oncology outpatient clinic in both institutes were recorded. Since it is easier to implement, the performance status of the patients was evaluated with ECOG performance scoring.

The present study was approved by the Manisa Celal Bayar University Faculty of Medicine ethics committee/institutional review board (approval No. E-85252386-050.04.04-49119, date: 22.03.2021) and the need of informed consent was waived by Manisa Celal Bayar University Faculty of Medicine ethics committee due to the retrospective study. We confirm that all methods were performed in accordance with relevant named guidelines and regulations.

During the evaluation of laboratory tests, CEA and CA19-9 were quantified from the peripheral blood samples of patients using the Cobas 6000 e 601 module (Roche Diagnostics, Switzerland) and Beckman Unicel DXL 800 (Beckman Coulter Diagnostics, United States) devices. Serum albumin, creatinine, lactate dehydrogenase (LDH), serum calcium and CRP levels were investigated using the Cobas 6000 c 501 Chemistry System (Roche Diagnostics, Switzerland) and Beckman AU 5800 Chemistry System (Beckman Coulter Diagnostics, United States). Hemogram tests (hemoglobin, platelet, neutrophil, lymphocyte) were performed using the Sysmex Xn-1000 (Sysmex Diagnostics, Japan) and Mindray BC 6800 (Mindray medical, China) devices. In both institutes, the units of the tests and reference ranges of the examined parameters were the same. The NLR and PLR values were calculated using the individual counts from peripheral blood. As in the original study, IPI was calculated using the formula: CRP × NLR/serum albumin.

OS was calculated from the date of metastatic disease diagnosis to the date of death or the date of last follow-up. PFS was calculated as the interval between the date of diagnosis and progression or death. Disease progression was accepted as imaging compatible with progressive disease on CT or PET-CT according to the response evaluation criteria in solid tumors version 1.1 (RECIST v1.1).

### Statistical analysis

Categorical variables are presented in tables as patient numbers and percentages. Comparisons were performed with odds ratios (OR) and corresponding 95% confidence intervals (CI) using chi-square or Fisher's exact test. Kaplan Meier analysis was used to depict survival curves. Univariate and multivariate analyzes were performed using the Cox proportional hazards model to assess the survival difference. Chi-square tests were used to analyze the relationship between NLR, PLR, IPI and clinicopathological parameters. Only variables that were significant as prognostic parameters in the univariate Cox's proportional hazards model were included in the multivariable analysis to identify independent prognostic factors. Variables which were added in the multivariate cox regression analysis were assessed with spearman rank correlation test to exclude multicollinearity. None of the bivariate correlation coefficients were above 0.7, which in turn indicated lack of significant multicollinearity. ROC analysis was used to determine the discriminative power of IPI score and to identify discriminatory cut-off. All statistical evaluations were a *p* value of 0.05 was considered statistically significant. All data were analysed using a commercially available software program (IBM SPSS statistics for windows, version 22.0, Armonk, NY: IBM corp).

### Ethical approval

Study was approved by the Manisa Celal Bayar University’s Non-Invasive Clinical Research Ethics Committee (approval No. E-85252386-050.04.04-49119, date: 22.03.2021).

### Patient consent

Since the study was a retrospective archive search, informed consent was not obtained from the patients.

## Results

A total of 152 patients were included for analysis in the present study. All patients had stage 4 disease. Median age at diagnosis was 63 years (range: 32–88). 30.9% (n = 47) of the patients were female, 69.1% (n = 105) were male. Demographic data are given in Table 1 and clinical features are given in Table 2.

**Table 1:** Demographic data and laboratuary findings of the stage-4 gastric cancer patients.

	Median (minimum–maximum) (n = 152)
Age	63 (32–88)
Height	165 (143–185)
Weight	62 (38–120)
CEA	4.275 (0.1–1744)
CA19-9	33 (0.5–9868)
Creatinine	0.775 (0.38–3.33)
Albumin	3.7 (2.08–4.7)
CRP	2.3 (0.11–29)
Neutrophil	5.84 (1.3–26)
Lymphocyte	1.52 (0.15–4.63)
Platelet	306.5 (34–1157)
Hemoglobin	11.4 (4.5–16.4)

**Table 2:** Clinical features of stage-4 gastric cancer patients.

	n	%		n	%
*Sex*			*Lung metastasis*		
Female	47	30.9	No	124	81.6
Male	105	69.1	Yes	28	18.4
*ECOG*			*Bone metastasis*		
0–1-2	129	84.9	No	125	82.2
3	23	15.1	Yes	27	17.8
*Surgery*			*LN metastasis*		
No	107	70.4	No	7	4.6
Yes	45	29.6	Yes	145	95.4
*Denovo*			*1st line*		
No	25	16.4	No	23	15.1
Yes	127	83.6	Yes	129	84.9
*Liver metastasis*			*Exitus*		
No	86	56.6	No	9	5.9
Yes	66	43.4	Yes	143	94.1

It was found that 83.6% (n = 127) of the patients had de novo metastatic disease. Twenty-three (15.1%) patients had an ECOG performance score of 3–4, and none of these patients received chemotherapy. The number of patients who received chemotherapy as first-line treatment was 129 (84.9%) and 67 (44.1%) patients received chemotherapy as second-line therapy. Overall, 143 (94.1%) of the patients had died at the time of data collection.

There were 45 (29.6%) patients who had previously undergone palliative or curative surgery for gastric cancer, while 107 (70.4%) were not operated. The number of Cerbb2 positive cases was 14 (9.2%). All of these cases received trastuzumab together with chemotherapy during first-line treatment.

Median PFS1 was 5.3 months, PFS2 was 3.3 months, and OS was 9.4 months. OS was 2.2 months in patients with ECOG 3–4, while it was 11 months in patients with ECOG 0–1-2 (*p* < 0.001). (mOS) is 11.9 months in patients with 2 or less metastatic areas, and 7.5 months in patients with 3 or more (*p* = 0.028).

The IPI value ranged from 0.2 to 38.7 (median, 22.2), and NLR ranged from 0.41 to 36.00 (median, 3.61).

**Figure 1:**
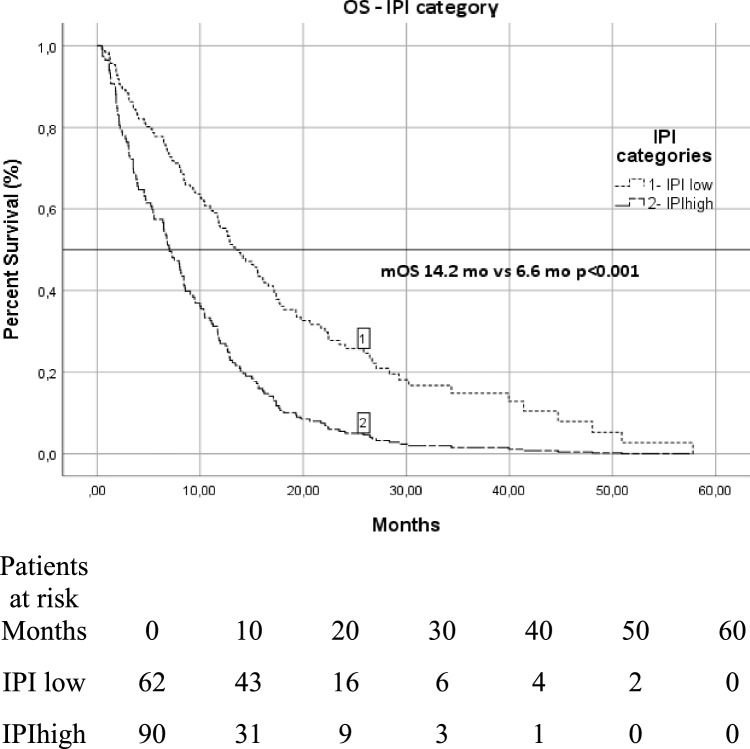
Overall survival patterns by IPI low and IPI high group.

**Figure 2:**
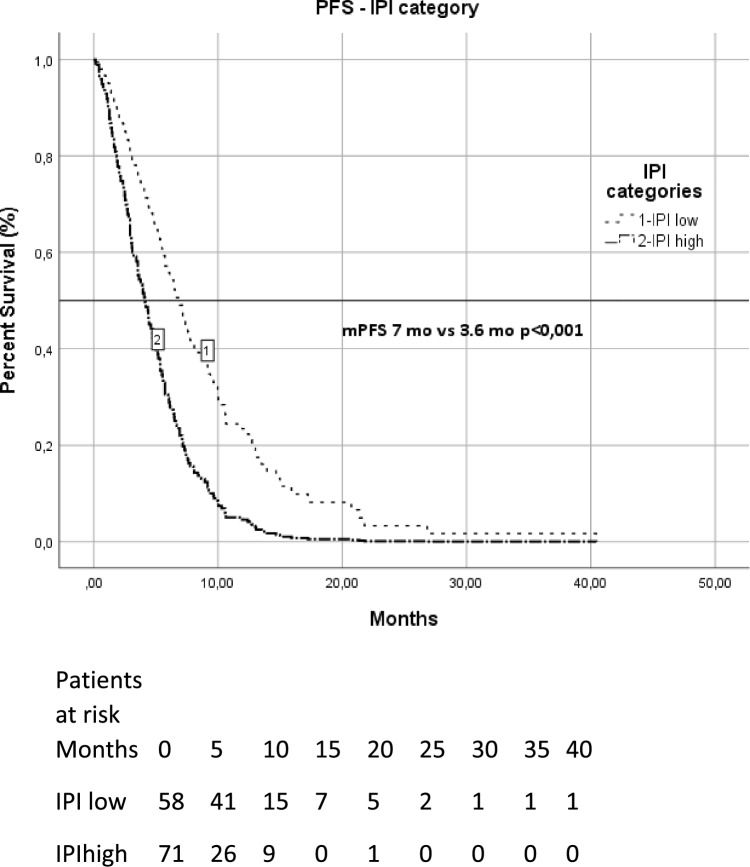
Progression free survival patterns by IPI low and IPI high group.

We evaluated IPI for its predictive value in survival status with ROC analysis and identified the cut-off value as 14.6. Low IPI score was significantly associated with longer OS and PFS compared to high IPI score (median OS in high vs. low IPI, 6.6 vs. 14.2 months; *p* < 0.001) (median PFS in high vs. low IPI, 3.6 vs. 7 months; *p* < 0.001). Survival function patterns by IPI low and IPI high groups are shown in Fig. 1. We evaluated NLR for its predictive value in survival status and identified the cut-off value as 3. Low NLR score was significantly associated with longer OS compared to high NLR score (median OS in high vs. low IPI, 7.2 vs. 13.6 months; *p* < 0.001) (median PFS in high vs. low NLR, 3,5 vs. 6.6 months; *p* < 0.001). Survival function patterns by NLR low and NLR high group has shown in Fig. 2. Notably, IPI classification were identified as an independent prognostic factor for OS and PFS.

The number of patients who received chemotherapy as a monotherapy (capecitabine, 5-FU, taxane) or doublet regimen (FOLFOX, XELOX, Platin-5-FU, FOLFIRI, Platin-Taxane, Taxane-5FU) was 59.7% (77), triplet regimen (FLOT, DCF, ECF) recipients represented 40.3% (52). While 42.9% (33) of 77 patients who received monotherapy or doublet regimen as first-line chemotherapy were in the IPI low group, 57.1% (44) were in the IPI high group. While 48.1% (25) of 52 patients who received triplet chemotherapy regimen were in the IPI low group, 51.9% (27) were in the IPI high group. There was no significant difference between the groups (*p* = 0.56).

While 48.1% (37) of 77 patients who received monotherapy or doublet regimen as first-line chemotherapy were in the NLR low group, 51.9% (40) were in the NLR high group. While 42.3% (22) of 52 patients who received triplet chemotherapy regimen were in the NLR low group, 57.7% (30) were in the NLR high group. There was no significant difference between the groups (*p* = 0.52).

While 53.2% (41) of 77 patients who received monotherapy or doublet regimen as first-line chemotherapy were in the PLR low group, 46.8% (36) were in the PLR high group. While 46.2% (24) of 52 patients who received triplet chemotherapy regimen were in the PLR low group, 53.8% (28) were in the PLR high group. There was no significant difference between the groups (*p* = 0.43).

Univariate and multivariable analyses of OS and PFS by prognostic factors are summarized in Tables 3 and 4.

**Table 3:** Univariate and multivariate analysis of overall survival by prognostic factors.

Characteristics	Univariate analysis		Multivariate analysis	
	OS HR (95%CI)	*P* value	OS HR (95%CI)	*P* value
IPI low versus high	0.45 (0.32–0.64)	< 0.001	0.39(0.25–0.59)	< 0.001
NLR low versus high	0.50 (0.35–0.70)	< 0.001	0.62(0.39–0.99)	0.045
Primary surgery	1.99 (1.37–2.9)	< 0.001	1.75(1.11–2.76)	0.016
Age ≤ 65 versus > 65	1.49 (1.05–2.11)	0.027	1.98(1.28–3.05)	0.002
ECOG 0–1–2 versus 3–4	0.51 (0.32–0.81)	0.004	0.58(0.34–0.79)	0.006
Recurrent versus De novo	0.55 (0.35–0.87)	0.01	0.56(0.31–1.02)	0.057
PLR low versus high	0.87 (0.62–1.21)	0.40	1.13(0.73–1.75)	0.590
CT M-D versus CT T	1.19 (0.83–1.72)	0.35	1.52(0.89–2.28)	0.056
Metastatic site 1–2 versus ≥ 3	0.78 (0.56–1.08)	0.13	0.85(0.59–1.24)	0.396
Sex male versus female	0.91 (0.63–1.3)	0.59	1.06(0.68–1.66)	0.793

*IPI* inflammatory prognostic index, *NLR* neutrophil/lymphocyte ratio, *PLR* platelet/lymphocyte ratio, *CT M-D* chemotherapy regimen monotherapy-doublet, *CT T* chemotherapy regimen triplet.

**Table 4:** Univariate and multivariable analysis of progression free survival by prognostic factors.

Characteristics	Univariate analysis		Multivariable analysis	
	PFS HR (95%CI)	*P* value	PFS HR (95%CI)	*P* value
IPI low versus high	0.47(0.33–0.68)	< 0.001	0.55(0.36–0.82)	0.004
NLR low versus high	0.50(0.35–0.72)	< 0.001	0.63(0.40–0.97)	0.04
Primary surgery	1.57(1.07–2.3)	0.02	0.71(0.47–0.07)	0.10
PLR Low versus High	0.85(0.6–1.2)	0.36	0.97(0.63–1.50)	0.90
Age ≤ 65 versus > 65	1.24(0.86–1.78)	0.26	0.71(0.46–1.08)	0.11
CT M-D versus CT T	1.05(0.74–1.50)	0.79	0.85(0.57–1.28)	0.44
Metastatic site 1–2 versus ≥ 3	0.92(0.65–1.3)	0.62	0.94(0.66–1.34)	0.72
Recurrent versus De novo	0.73(0.44–1.23)	0.24	0.87(0.49–1.54)	0.63
Sex male versus female	0.87(0.59–1.28)	0.48	1.19(0.76–1.86)	0.46

*IPI* inflammatory prognostic index, *NLR* neutrophil/lymphocyte ratio, *PLR* platelet/lymphocyte ratio, *CT M-D* chemotherapy regimen monotherapy-doublet, *CT T* chemotherapy regimen triplet.

## Discussion

Three inflammatory markers are very important for prediction of survival in gastric cancer. These are NLR, CRP and serum albumin level^13^. There are several prognostic marker systems that allow the interpretation of prognosis based on inflammation in solid malignant tumors. As mentioned, high NLR has been identified as an unfavorable prognostic factor in various cancers. IL-1, IL-6 and tumor necrosis factor-α are at the root of the increase in CRP, an acute phase inflammation protein. CRP is also known to contribute to aggressive cancer behavior, and it has been established as a factor of poor prognosis in various solid tumor types^14^.

Hypoalbuminemia may be another important variable as it has been shown to be significantly associated with shorter survival time in cancer patients. Hypoalbuminemia and high CRP levels are frequently observed in advanced cancer patients and are generally reported to be associated with worse survival^15^. This may be associated with the fact that protein digestion and absorption are decreased in patients with gastric cancer, resulting in a negative nitrogen balance^16^. For this reason, it is thought that the IPI, which is the combination of these three important parameters, can serve as a more effective scoring system in predicting the prognosis of cancer patients. A higher IPI score indicates more severe inflammation and a weaker immune response in patients.

In this analysis of 152 gastric cancer patients, we found that NLR and IPI were associated with survival status, as measured by OS and PFS. The IPI is a reproducible, cost-effective and available prognostic marker for gastric cancer patients^17^. Several prognostic scores have been developed to aid in the assessment of cancer prognosis, some of which are based on serum CRP and albumin levels, such as the Glasgow prognostic score. Similar to IPI, Glasgow prognostic score scores demonstrating high inflammation and poor immune response have been shown to be associated with worse prognosis in a variety of different tumor types^12,18^. We propose that patients with a high NLR and high IPI score should thus be recognized as a high-risk group in terms of progression and survival.

An important previously published study demonstrated that CRP/albumin ratio and NLR serve as independent prognostic factors for OS in patients with gastric cancer^19^. In the present study, Cox regression analysis demonstrated that the IPI score could be used as a predictor for both OS and PFS. There are studies showing that inflammatory indices such as NLR and systemic immune inflammation index can predict treatment response and are associated with chemotherapy resistance^20,21^. IPI similarly appears to be able to predict both treatment response and OS since IPI was associated with PFS and OS regardless of the preferred chemotherapy regimen. Also, IPI seems to be a more sensitive index compared to NLR in the prediction of PFS and OS.

Since this study was a retrospective two-center study with a relatively small number of patients, it would be appropriate to confirm these results with prospective studies with larger patient participation.

## Conclusion

As a result of cox regression analysis, it was determined that IPI score could be a parameter that could predict prognosis and survival in patients with metastatic gastric cancer. The IPI score can be an independent prognostic index that is inexpensive, easy to access and evaluate for metastatic gastric cancer patients, and may be useful in predicting survival in daily clinical practice.

## Supplementary Information

Supplementary Information.

## Data Availability

The datasets used and/or analysed during the current study available from the corresponding author on reasonable request.
